# Supplementary material for: Recent and rapid reef recovery around Koh Phangan Island, Gulf of Thailand, driven by plate-like hard corals
Source: PeerJ. 2023 Nov 9;11:e16115. doi: 10.7717/peerj.16115 (PMC10640840; doi:10.7717/peerj.16115)
Supplement: Supplemental Information 1 [file peerj-11-16115-s001.docx]

**Recent and rapid reef recovery around Kho Phangan Island, Gulf of Thailand, driven by plate-like hard corals**

**- Supplementary Material -**

Florian Stahl^1,2,°^, Selma D. Mezger^1,°,*^, Valentina Migani^3^, Marko Rohlfs^4^, Victoria J. Fahey^5^, Eike Schoenig^5^, Christian Wild^1^

^1^University of Bremen, Faculty of Biology and Chemistry, Marine Ecology Group, Leobener Str. 6, 28359 Bremen, Germany

^2^University of Bremen, Faculty of Biology and Chemistry, Marine Botany Group, Leobener Str. 5, 28359 Bremen, Germany

^3^University of Bremen, Faculty of Biology and Chemistry, Evolutionary Biology Group, Leobener Str. 5, 28359 Bremen, Germany

^4^University of Bremen, Faculty of Biology and Chemistry, Chemical Ecology Group, Leobener Str. 5, 28359 Bremen, Germany

^5^Center for Oceanic Research and Education (COREsea), Chaloklum, Koh Phangan, Thailand

° shared first authorship

* corresponding author: Selma D. Mezger, [mezger@uni-bremen.de](mailto:mezger@uni-bremen.de)

**Supplementary Material Table S1**. Four coral growth forms (massive, branching, plate-like, and solitary) of Koh Phangan and their assigned hard coral genera.

| **Massive** | **Branching** | **Plate-like** | **Solitary** | |
| --- | --- | --- | --- | --- |
|  |  |  |  | |
| *Lobophyllia* | *Acropora* | *Montipora* | *Heliofungia* | |
| *Astreopora* | *Pocillopora* | *Leptoseris* | *Fungia* | |
| *Porites* | *Seriatopora* | *Lithophyllon* | *Ctenactis* | |
| *Goniopora* | *Stylophora* | *Turbinaria* | *Sandalolitha* | |
| *Galaxea* |  | *Pachyseris* |  | |
| *Diploastrea* |  | *Merulina* |  | |
| *Dipsastraea* |  | *Echinophyllia* |  | |
| *Goniastrea* |  | *Pavona* |  | |
| *Alveopora* |  | *Podabacia* |  | |
| *Leptoria* |  | *Pectinia* |  | |
| *Favites* |  | *Mycedium* |  | |
| *Leptastrea* |  |  |  | |
| *Acanthastrea* |  |  |  | |
| *Oulophyllia* |  |  |  |  |
| *Physogyra* |  |  |  |  |
| *Coeloseris* |  |  |  |  |
| *Platygyra* |  |  |  |  |
| *Gardineroseris* |  |  |  |  |
| *Cyphastrea* |  |  |  |  |
| *Echinopora* |  |  |  |  |
| *Euphyllia* |  |  |  |  |
| *Plerogyra* |  |  |  |  |
| *Coscinaraea* |  |  |  |  |
| *Psammocora* |  |  |  |  |
| *Hydnophora*  *Plesiastrea* |  |  |  |  |
| *Astrea* |  |  |  |  |

**Supplementary Material Table S2**. Coral genera of Koh Phangan and their appearance in the different years of surveying the reefs. “x” represents the genus being present along transects that year, while “-“ indicate the genus missing along transects that year.

| **Genus** | | **2014** | **2015** | **2016** | **2017** | **2018** | **2019** | **2022** |
| --- | --- | --- | --- | --- | --- | --- | --- | --- |
| ACANTHASTREA | | x | x | x | x | x | x | x |
| ACROPORA | | x | x | x | x | x | x | x |
| ALVEOPORA | | x | x | x | x | x | x | x |
| ASTREOPORA | | x | x | x | x | x | x | x |
| CAULASTREA | | - | - | - | - | - | - | - |
| COELOSERIS | | - | x | - | x | x | x | x |
| COSCINARAEA | | - | x | - | - | x | - | - |
| CTENACTIS | | - | - | x | - | x | x | - |
| CYNARINA | | - | - | - | - | - | - | - |
| CYPHASTREA | | - | - | x | x | x | x | x |
| DIPLOASTREA | | x | x | x | x | x | x | x |
| ECHINOPHYLLIA | | - | x | - | x | x | - | x |
| ECHINOPORA | | x | x | x | x | x | x | x |
| EUPHYLLIA | | x | x | x | x | x | x | - |
| FAVIA | | - | - | - | - | - | - | - |
| FAVITES | | x | x | x | x | x | x | x |
| FUNGIA | | x | x | x | x | x | x | x |
| GALAXEA | | - | x | x | x | x | x | x |
| GARDINEROSERIS | | x | - | x | x | - | x | - |
| GONIASTREA | | x | x | x | x | x | x | x |
| GONIOPORA | | x | x | x | x | x | x | x |
| HELIOFUNGIA | | - | x | - | - | - | - | - |
| HYDNOPHORA | | - | - | - | x | x | x | x |
| LEPTASTREA | | - | - | - | x | - | x | x |
| LEPTORIA | | - | x | x | - | x | x | - |
| LEPTOSERIS | | x | x | x | x | x | x | x |
| LITHOPHYLLON | | - | x | x | x | x | - | x |
| LOBOPHYLLIA | | x | x | x | x | x | x | x |
| MERULINA | | - | x | x | x | x | x | x |
| MONTASTREA | | - | - | - | - | - | - | - |
| MONTIPORA | | x | x | x | x | x | x | x |
| OULOPHYLLIA | | - | x | x | x | x | x | x |
| PACHYSERIS | | x | x | x | x | x | x | x |
| PAVONA | | x | x | x | x | x | x | x |
| PECTINIA | | - | - | - | x | x | x | x |
| PHYSOGYRA | | - | - | - | - | x | x | - |
| PLATIGYRA | | - | - | - | - | - | - | - |
| PLEROGYRA | | x | x | x | x | x | x | x |
| POCILLOPORA | | x | x | x | x | x | x | x |
| PODABACIA | | - | - | - | - | - | - | - |
| PORITES | | x | x | x | x | x | x | x |
| PSAMMOCORA | | - | - | - | x | x | - | - |
| SANDALOLITHA | | - | - | - | - | - | - | x |
| SERIATOPORAA | | - | - | - | - | - | - | - |
| STYLOPHORA | | x | x | - | - | x | - | - |
| SYMPHYLLIA | | - | - | - | - | - | - | - |
| TUBASTREA | - | | - | - | - | - | - | - |
| TURBINARIA | | x | x | x | x | x | x | x |
| MYCEDIUM | | - | - | - | - | - | - | x |
| SERIATOPORA | | - | - | - | - | x | x | - |
| PLESIASTREA | | - | - | - | - | - | - | x |
| ASTREA | | - | x | - | x | x | x | x |
| DIPSASTRAEA | | x | x | x | x | x | x | x |
| PLATYGYRA | | x | x | x | x | x | x | x |
| PODOBACIA | | - | x | x | x | x | x | x |

**Supplementary Material Table S3**. Total number of transects that were conducted each year.

| **YEAR** | **Transects** |
| --- | --- |
| 2011 | 6 |
| 2012 | 10 |
| 2013 | 11 |
| 2014 | 18 |
| 2015 | 61 |
| 2016 | 78 |
| 2017 | 152 |
| 2018 | 162 |
| 2019 | 104 |
| 2020 | 10 |
| 2021 | 0 |
| 2022 | 117 |

**Supplementary Material Table S4**. Months per year, during which transects were carried out. “x” indicate that transects were conducted in that month.

| **YEAR** | **Jan** | **Feb** | **Mar** | **Apr** | **May** | **Jun** | **Jul** | **Aug** | **Sep** | **Oct** | **Nov** | **Dec** |
| --- | --- | --- | --- | --- | --- | --- | --- | --- | --- | --- | --- | --- |
| **2011** |  | x | x |  |  |  |  |  |  |  |  |  |
| **2012** |  |  |  |  |  |  |  | x | x |  | x |  |
| **2013** |  |  |  |  | x |  | x | x |  |  |  |  |
| **2014** |  |  | x | x |  |  |  | x | x |  |  |  |
| **2015** | x |  | x | x | x | x | x | x | x |  | x |  |
| **2016** | x | x | x | x | x | x | x | x | x | x |  |  |
| **2017** | x | x | x | x | x | x | x | x | x |  |  |  |
| **2018** | x | x | x | x | x | x | x | x | x | x | x |  |
| **2019** | x | x | x | x | x | x | x | x | x | x | x |  |
| **2020** |  | x | x |  |  |  |  |  |  |  |  |  |
| **2021** |  |  |  |  |  |  |  |  |  |  |  |  |
| **2022** |  |  | x | x | x | x | x | x | x | x | x |  |

**COREsea long-term monitoring background information of main reef sites**

All reef sites are fringing reefs with a general topographic composition of 1-2m reef flat, 3-6m reef crest which slopes down to the forereef with the reef edge at a max. depth of 10m.

**Ao Thong Lan – ATL**

Reef coordinates (lat/lon):

| 9.799615 | 99.988284 |
| --- | --- |

- Low tourism impact
- Less developed area compared to MH4, HY, HS, KM
- In recent years influenced by pulse sedimentation events when it rains. This comes from villa construction close to the reef
- Affected by “severe bleaching” (> 50% coral colonies bleached) in 2014, 2015, 2016, 2019

**Ao Chaloklum (bay east) – CBE**

Reef coordinates (lat/lon):

| 9.796695 | 100.012326 |
| --- | --- |

- Chaloklum Bay is a large bay located in the north of Koh Phangan, however, we survey a fringing reef located on the east side
- Chaloklum is highly developed with historically a lot of coastal development. There are two manmade seawalls on the west and east side of the bay protecting the coast
- There is relatively large “klong” or canal that drains into the east side of Chaloklum used mainly for longtail boat traffic. Longtails park in the klong when there is bad weather
- This bay has a lot of boat traffic and is a hotspot for dive boats, longtail boats, and fishing vessels that moor up for shelter
- During northeast monsoon season (November/December), this area gets a lot of mechanical damage on the reef due to large storm waves
- Affected by “severe bleaching” (> 50% coral colonies bleached) in 2018, 2019

**Haad Khom – HK**

Reef coordinates (lat/lon):

| 9.798695 | 100.01505 |
| --- | --- |

- Moderate coastal development
- High tourism impact – during high season it’s a popular spot for snorkel tours, and larger tourism boat operators also moor up into the bay. Can see a lot of mechanical damage from boats during high season
- Usually more turbid than other reef sites
- Affected by “severe bleaching” (> 50% coral colonies bleached) in 2014, 2016, 2018, 2019

**Haad Salad – HS**

Reef coordinates (lat/lon):

| 9.786756 | 99.970869 |
| --- | --- |

- Highly developed coastal area with lots of resorts and restaurants on the beach
- High tourism impact – popular spot for snorkel tours
- Steeper built up reef crest compared to other sites

**Haad Yao – HY**

Reef coordinates (lat/lon):

| 9.775804 | 99.964428 |
| --- | --- |

- Highly developed coastal area with lots of resorts and restaurants on the beach
- Medium tourism impact – not so popular with snorkel tours but frequented on occasion with some dive schools
- Steeper built up reef crest compared to other sites
- Although on our main list of sites we visit for our long-term monitoring program, this is the reef we visit the least, mainly due to distance – therefore we have patchy replicates for surveys and for some years not even visited at all

**Koh Ma – KM**

Reef coordinates (lat/lon):

| 9.801755 | 99.977899 |
| --- | --- |

- Koh Ma is a small island attached by a sand bar to Mae Haad beach
- Part of the Than Sadet National Park and is under a protected status from fishing, however, this isn’t enforced
- There is a large reef system that stretches from around Koh Ma and heads south to the end of Mae Haad which we call MH4. However, MH4 is distinguished as a separate reef because there is a large break in the reef caused by a manmade channel for longtail boats to come in and out of the shore
- Highly developed coastal area with resorts on the beach
- High tourism impact – very popular for snorkelling/diving tours, in general a very popular beach for tourism
- Steeper built up reef crest compared to other sites
- Prone to mechanical damage from boat traffic – tourism operators not mooring up to buoys provided and dropping anchor straight onto the reef
- The reef around here is exposed to currents compared to other sites

**Mae Haad 4 – MH4**

Reef coordinates (lat/lon):

| 9.79226 | 99.976231 |
| --- | --- |

- In general, Mae Haad is a very large bay which we have categorised into 5 distinct ecological zones mainly based on differences in coral cover and topography
- MH4 is located at the southern end of Mae Haad Bay with a beach in front of the reef that has no coastal development
- Due to the hydrodynamics of the bay, MH4 is heavily affected by pulse sedimentation events where a small river inlet runs down into the bay where sediments from coastal development run into the reefs when heavy rains occur
- April, May, June of 2017 there was a large sedimentation event from heavy rainfall and recent construction where sediments smothered corals in the reef. Main corals affected were plating Pachyseris
- Low tourism impact – no one frequents this side of Mae Haad other than us


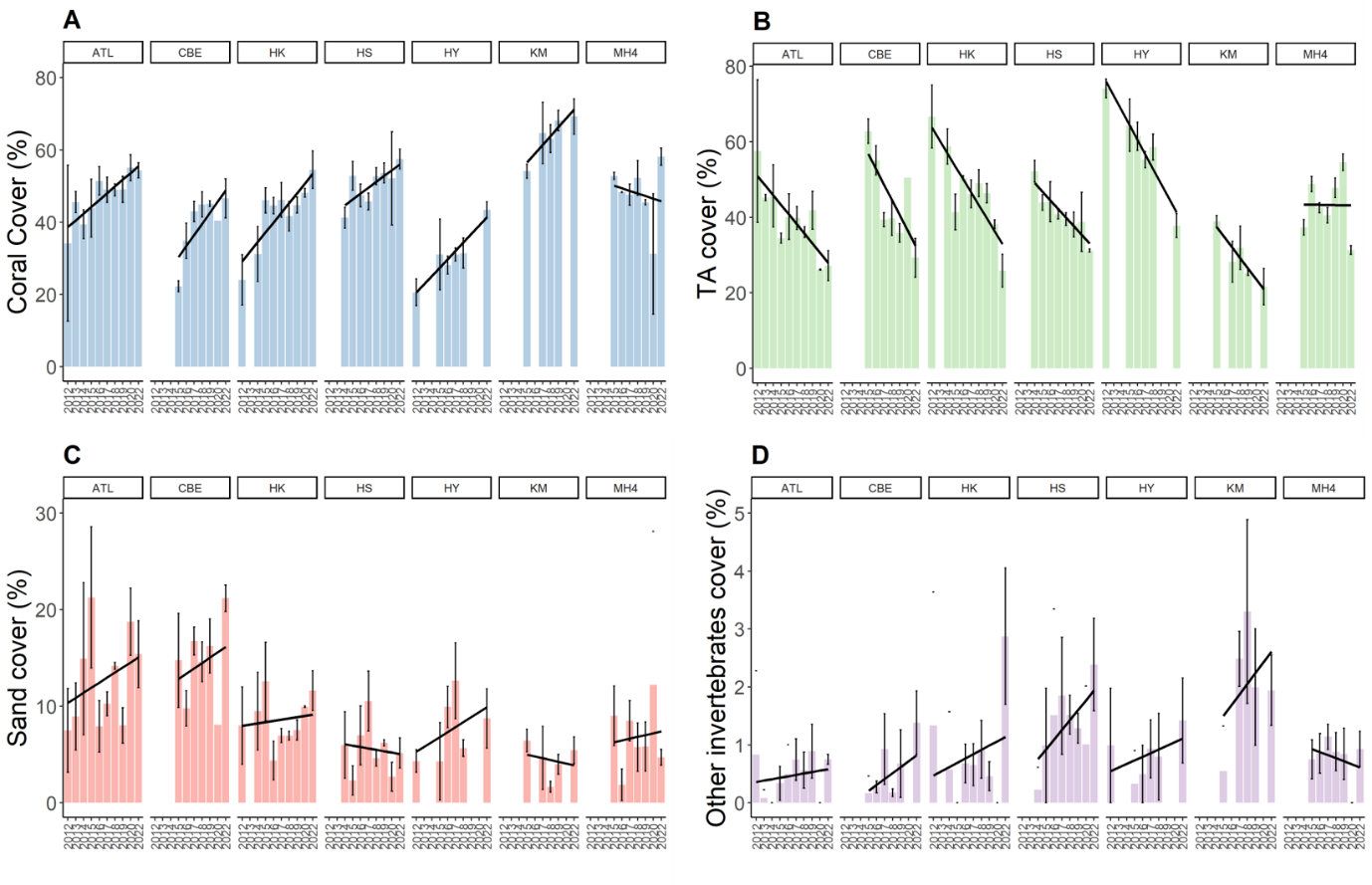


**Supplementary Material Figure S1.** Development of the benthos cover from 2012 to 2022, separated by reef sites. (A) Live coral cover, (B) turf algae, (C) sand cover, and (D) other invertebrates cover. Bars represent the mean and error bars the standard error of the cover in the study area, while black lines represent the linear regression lines.


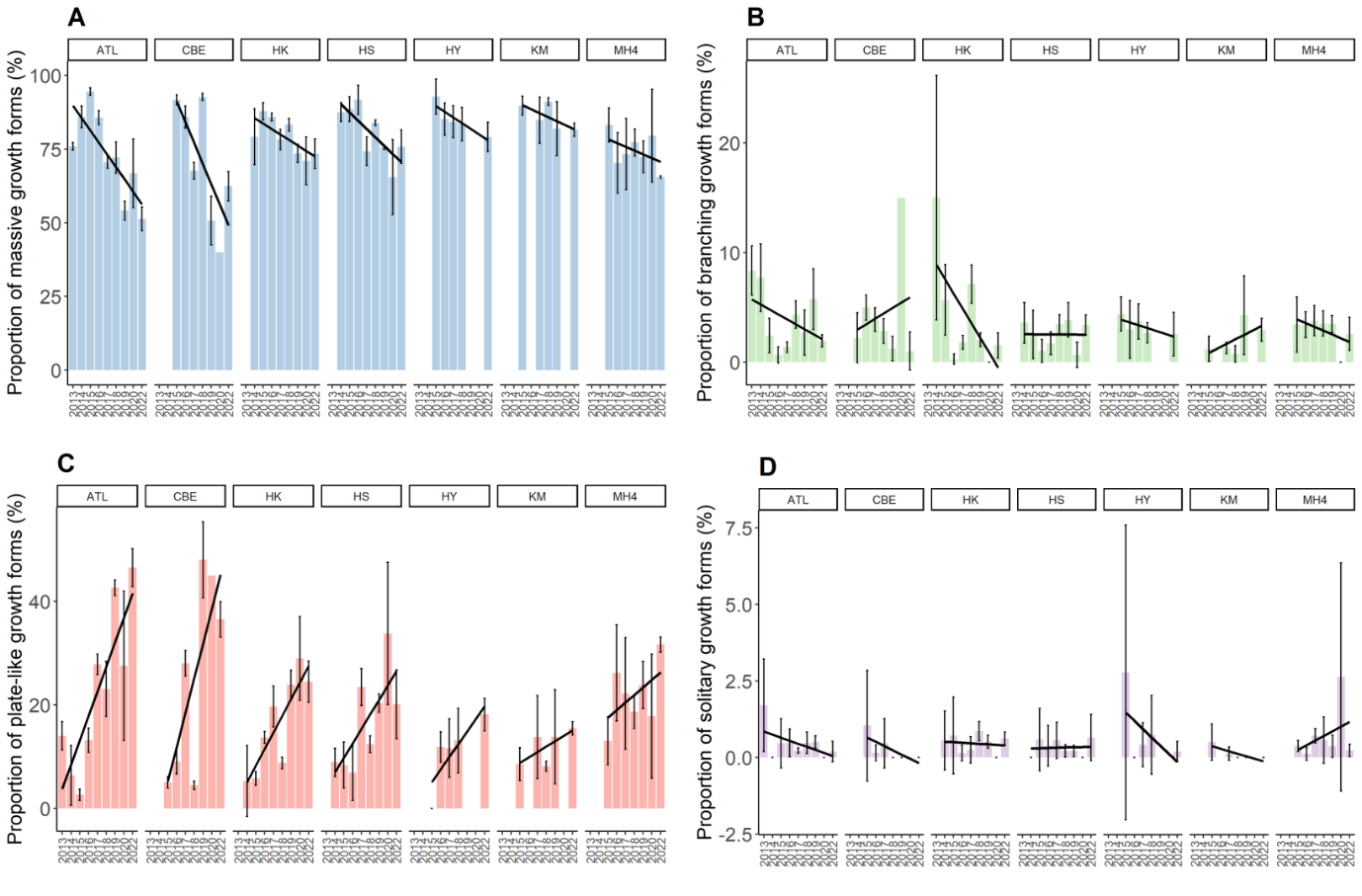


**Supplementary Material Figure S2.** Percent coverage of growth forms from 2013 to 2022, separated by reef sites. (A) Massive growth forms, (B) branching growth forms, (C) plate-like growth forms, and (D) solitary growth forms. Bars represent the mean and error bars the standard error of the cover in the study area, while black lines represent the linear regression lines.


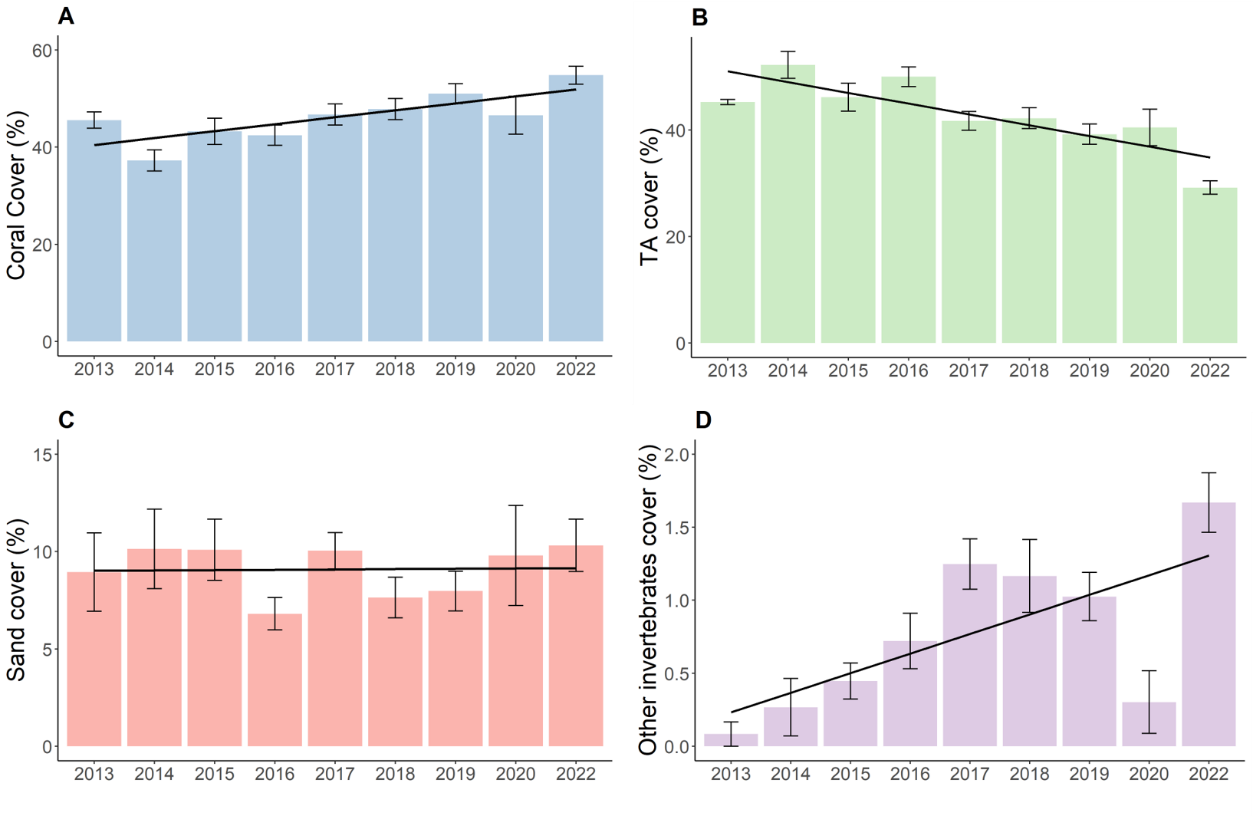


**Supplementary Material Figure S3.** Development of the benthos cover from 2013 to 2022. (A) Live coral cover, (B) turf algae, (C) sand cover, and (D) other invertebrates cover. Bars represent the mean and error bars the standard error of the cover in the study area, while black lines represent the linear regression lines.


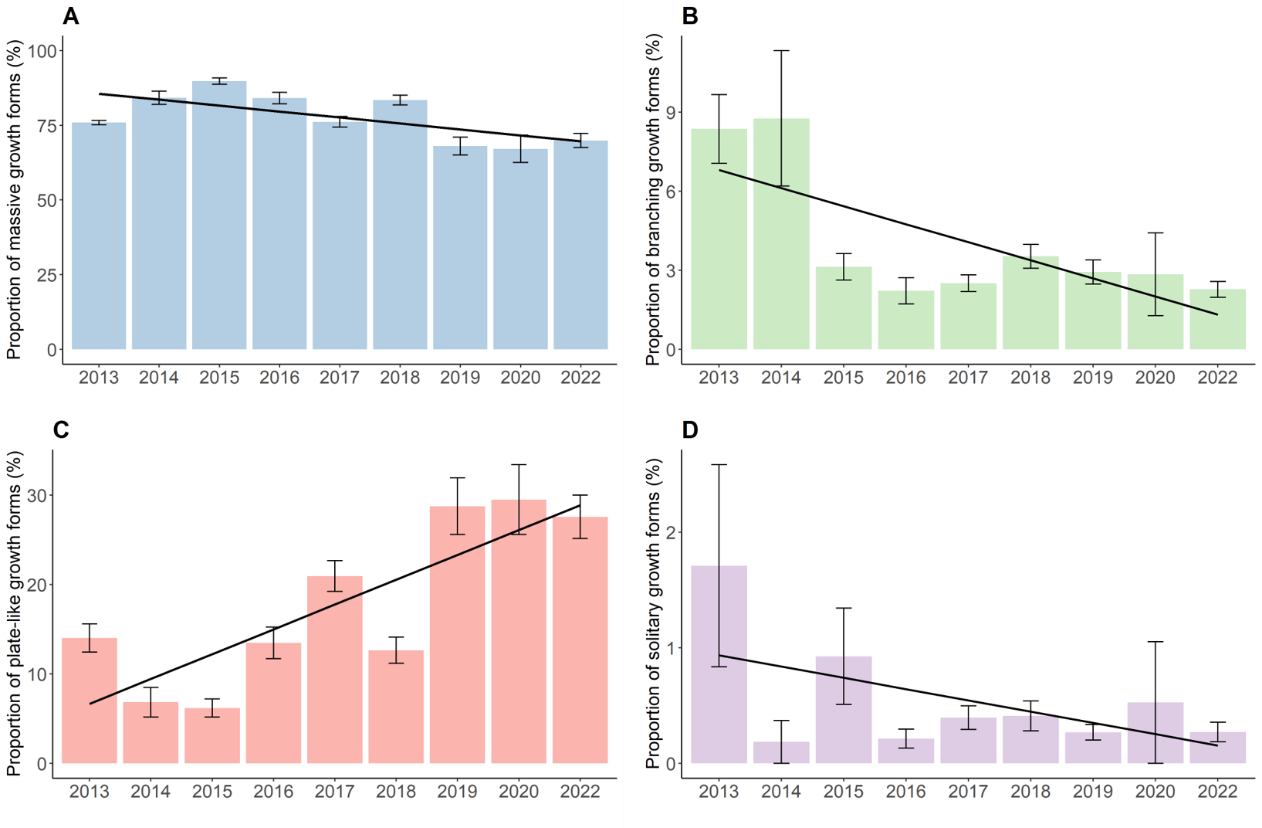


**Supplementary Material Figure S4.** Percent coverage of growth forms from 2013 to 2022. (A) Massive growth forms, (B) branching growth forms, (C) plate-like growth forms, and (D) solitary growth forms. Bars represent the mean and error bars the standard error of the cover in the study area, while black lines represent the linear regression lines.

**Bleaching around Koh Phangan**


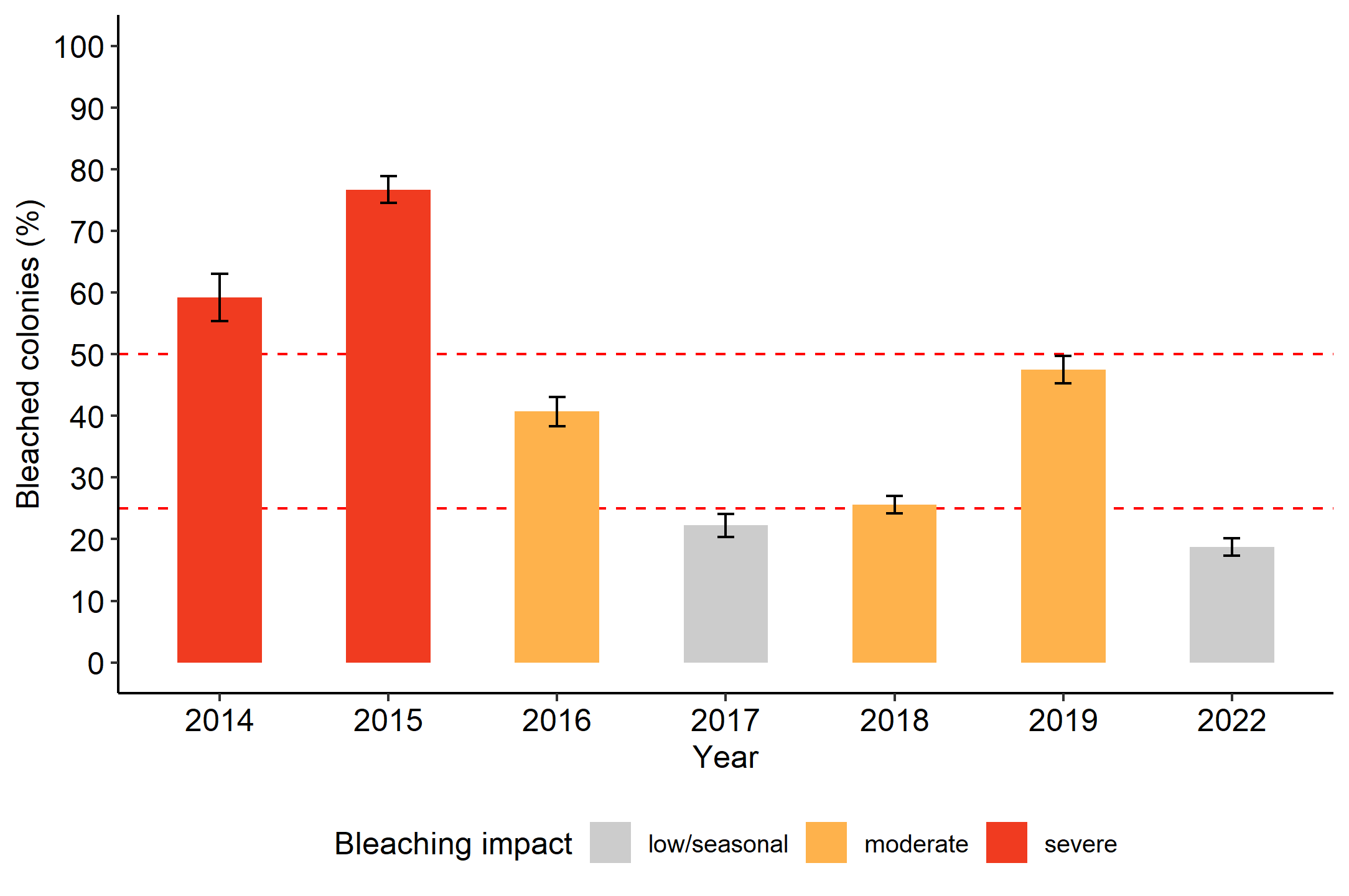


**Supplementary Material Figure S5.**  Percent coral colonies being either fully or partially bleached within the hottest months of May until July in the years 2014 to 2022. Bars represent the mean and error bars the standard error of the bleaching in the study area.


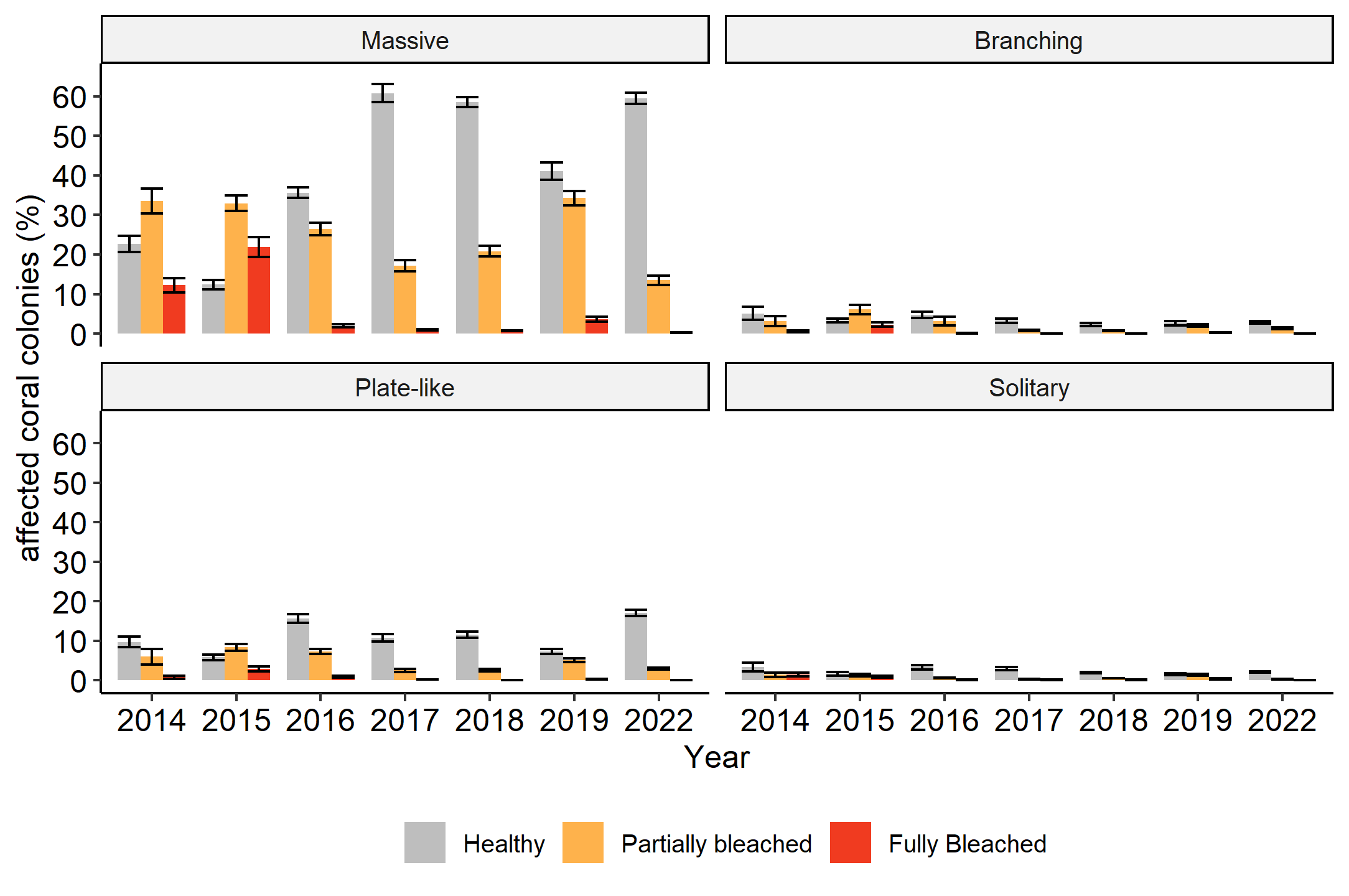


**Supplementary Material Figure S6.**  Percent of growth forms being healthy, partially bleached, or fully bleached within the hottest months of May until July in the years 2014 to 2022. Bars represent the mean and error bars the standard error of the cover in the study area.
